# Editorial: Novel anti-cancer agents targeting tumour metastasis and stemness, volume II

**DOI:** 10.3389/fphar.2026.1740454

**Published:** 2026-03-11

**Authors:** Bo Wang, Luping Pang

**Affiliations:** 1 Key Laboratory of Advanced Drug Preparation Technologies, Ministry of Education, Zhengzhou, China; 2 School of Pharmaceutical Sciences, Zhengzhou University, Zhengzhou, China; 3 Department of Medical Genetics and Cell Biology, School of Basic Medical Sciences, Zhengzhou University, Zhengzhou, China

**Keywords:** cancer stem cells, novel anti-cancer strategy, signaling pathway, tumor metastasis, tumor relapse

Tumor stemness is the core biological property of cancer stem cells (CSCs), manifested in their abilities for self-renewal, differentiation, and evasion of immune surveillance and drug-induced killing. CSCs are proposed as “seeds” of tumors and are the key drivers of tumor initiation, progression, and recurrence. During tumor initiation, CSCs bypass physiological growth control boundaries by accumulating oncogenic mutations, thereby initiating tumor formation (Prasad et al., 2020). In the progression stage, their differentiation ability maintains tumor cell heterogeneity, allowing the tumor to adapt to aggressive microenvironments, such as hypoxia and nutrient deprivation. More importantly, CSCs are highly correlated with therapeutic resistance through upregulating drug efflux pumps and activating DNA repair pathways, making them a major reason for the failure of radiotherapy and chemotherapy. Tumor stemness is particularly closely related to metastasis. CSCs acquire migratory and invasive abilities through epithelial-mesenchymal transition (EMT), penetrating the extracellular matrix and blood vessel walls to enter the bloodstream. Upon reaching distant organs, their stemness characteristics facilitate disseminated tumor cells in surviving, proliferating, and forming metastatic lesions in new microenvironments (Testa et al., 2020). This “stemness-metastasis” synergy directly determines the therapeutic efficacy and prognosis of patients and is a major challenge in clinical cancer treatment.

Currently, the therapeutics targeting tumor stemness and metastasis are mainly divided into three categories. The first group mainly consists of signaling pathway inhibitors, such as inhibitors targeting CSC self-renewal regulatory pathways like Wnt, Notch, and Hedgehog signaling pathways, which block the core survival signals of CSCs (Bhal and Kundu, 2023). The second type includes EMT inhibitors that reduce the conversion of CSCs to invasive phenotypes by inhibiting pathways such as TGF-β/Smad and PI3K/AKT. The last group is immune-targeted drugs, such as monoclonal antibodies against reported CSC surface targets (CD44, CD133, ALDH1, and so on), guiding the immune system to specifically eliminate CSCs (Dai et al., 2025). However, off-target effects, rapid emergence of drug resistance, and drug delivery Research Topic of these therapeutics are still major challenges for clinical anti-metastasis and stemness treatment. This special edition aims to systematically explore the molecular mechanisms regulating tumor stemness and the “stemness–metastasis” interplay, to further deepen the understanding of their synergistic roles in tumor progression, thereby identifying new therapeutic targets, discovering potential anticancer drugs, developing novel drug delivery systems and evaluating the translational potential of these candidate therapies.

Osteosarcoma is a primary malignant bone tumor with a high incidence in adolescents, with high recurrence and metastasis rates. In addition, due to chemotherapy resistance and the lack of effective therapeutic drugs, metastatic patients show relatively low survival rates. Wei et al. primarily investigate the specific targets and molecular mechanisms of Solasonine, the main active component of the traditional Chinese medicine Long Kui, for combating osteosarcoma. The study innovatively integrates network pharmacology and transcriptomics techniques along with experimental validation. By screening differentially expressed genes, performing intersection analysis of candidate targets, and conducting regression analysis, five key potential targets (ATP1A1, CLK1, SIGMAR1, PYGM, HSP90B1) of Solasonine against osteosarcoma were ultimately identified. Prognostic models and nomograms constructed based on these targets can effectively assess patient prognosis. Moreover, related mechanisms were elucidated through pathway enrichment, immune microenvironment regulation, and molecular docking analysis. Experiments further confirmed that these key targets are highly expressed in osteosarcoma cells and that Solasonine can inhibit the malignant biological behaviors of these cells. This study provides a reference paradigm for the modernization of traditional Chinese medicine, and further mechanistic validation is expected to further promote the clinical translation of these findings.

Breast cancer brain metastasis (BC-BM) is one of the most severe complications of breast cancer. Current therapies for BC-BM, including temozolomide, have limited efficacy due to restricted drug penetration across the blood-brain barrier. To tackle this Research Topic, Feng et al. systematically explored the interaction mechanism between BC cells and the brain microenvironment via combinational mouse models and molecular biology techniques. The study found that M2-type microglia were significantly enriched in BC-BM lesions. The IL6 secreted by these cells reversed the mesenchymal-epithelial transition (MET) progression of BC cells by activating the JAK2/STAT3 signaling pathway to promote brain colonization, while CCL2 recruited monocytic myeloid-derived suppressor cells (M-MDSCs) to establish an immunosuppressive microenvironment. BC-BM cells themselves exhibited a mesenchymal phenotype and activated the JAK2-STAT3 pathway. However, β-elemene, the main active component of the traditional Chinese medicine *Curcumae Rhizoma*, significantly reduced the incidence of BC-BM in intracarotid model mice without obvious toxicity. This antitumor effect was mediated by inhibition of the IL6/STAT3 signaling pathway and reduced recruitment of M-MDSCs, and β-elemene showed better antitumor efficacy than temozolomide. This study for the first time clarified that M2-type microglia synergistically regulate the “colonization-immunosuppression” stages of BC-BM through IL6/CCL2 dual factors. By combining traditional Chinese medicine components with modern molecular approaches, this study elucidates the molecular basis of the “seed-soil” theory in BC-BM. It further highlights β-elemene as a low-toxicity therapeutic candidate and identifies potential targets, including the IL6/CCL2/STAT3 axis and M-MDSCs, providing a framework to overcome current treatment bottlenecks and improve patient outcomes.

Cancer stem cell-mediated drug resistance, recurrence, and metastasis are key factors contribute to clinical tumor therapeutic failure. To address this critical bottleneck in tumor treatment, as well as the high toxicity and emerging resistance of existing metastasis-targeted drugs, Xie et al. provided a comprehensive review of the development and therapeutic strategies of various novel drugs with a great translational potential. The authors described Thiolatia (THL) in detail. THL is a PSMD14-specific inhibitor that reverses EMT by inhibiting PSMD14-mediated SNAIL deubiquitination and disrupts cytoskeletal remodeling, thereby exerting anti-metastatic effects. Moreover, it enhances cancer cell sensitivity to cisplatin, demonstrating potent chemo-sensitizing activity with high selectivity and reduced toxicity compared with conventional proteasome inhibitors. Sulfarotene, which specifically targets liver cancer tumor-reconstructive cells (TRCs) via the RARα-SOS2-RAS axis to overcome sorafenib resistance, has shown low-toxicity. The review also introduces HA-NPs (hyaluronic acid-modified nanoparticles) for delivering the BMI-1 inhibitor PTC 209, addressing poor drug solubility and non-specific distribution in colorectal cancer while reversing stemness of CSCs. In addition, platinum derivatives were explored to conquer resistance through a “dual-targeting” mechanism. While Cantharidin, isolated from traditional Chinese medicine, provides multi-target anti-metastatic effects by regulating pathways such as PI3K/AKT/mTOR and MAPK, its toxicity remains to be addressed. This review highlights the potential applications in precision oncology, providing diverse solutions to overcome current treatment bottlenecks and offering a reference paradigm for “target-mechanism-delivery” integrated design in novel anti-tumor drug development. It also emphasizes the key importance of clinical validation for drug translation, laying a foundation for implementing precision therapy and improving cancer patient prognosis.

In summary, this Research Topic not only elucidates the key factors involved in different tumor metastases and stemness-targeted therapies, but also provides preclinical data and early clinical results supporting the treatment of these diseases through developing targeted drugs. The publications included in this Research Topic exemplify the core directions for future tumor metastasis and stemness therapy: including developing combination treatment strategies simultaneously blocking stemness and metastasis pathways, constructing precision treatment based on specific biomarkers, and optimizing drug delivery technologies to improve target enrichment efficiency. Ultimately, this Research Topic aims to build a bridge between basic research and clinical application, offering new ideas and hope for conquering metastatic cancers.
